# Mesonephric-like adenocarcinoma of the ovary: A case report and a review of the literature

**DOI:** 10.1097/MD.0000000000023450

**Published:** 2020-11-25

**Authors:** Qiuhe Chen, Yangmei Shen, Chuan Xie

**Affiliations:** a Department of Gynecology and Obstetrics, West China Second University Hospital; b Key Laboratory of Birth Defects and Related Diseases of Women and Children, Ministry of Education; c Department of Pathology, West China Second University Hospital, Sichuan University, Chengdu, Sichuan Province, China.

**Keywords:** case report, mesonephric adenocarcinoma, mesonephric-like adenocarcinoma, ovary, treatment

## Abstract

**Rationale:**

Mesonephric-like adenocarcinoma (MLA) from ovary is a very rare tumor which derives from mesonephric duct remnant of the female genital tract. Only six cases have been reported so far in the English literature.

**Patient concerns:**

A 29-year-old female patient was referred to the local hospital with a 20-day history of abdominal discomfort.

**Diagnoses:**

Pelvic ultrasound examination revealed a solid and cystic mass measuring 10 cm in diameter in the right adnexal area and a cystic mass measuring 5 cm in the left adnexal area. Postoperative pathology in the local hospital revealed suspected malignancy of the right ovary, and she was then transferred to our institution for definite diagnosis. The tumor mass was finally diagnosed as a primary MLA arising from the right ovary by histological and immunohistochemical examination in our institution.

**Interventions:**

The patient underwent laparoscopic right adnexectomy and removal of left ovarian cyst in the local institution. Then, she underwent a complete staging surgery including a total hysterectomy, left adnexectomy, pelvic plus para-aortic lymphadenectomy, and omentectomy in our hospital. In addition, she received four cycles of combination chemotherapy with carboplatin plus paclitaxel.

**Outcomes:**

There is no evidence of recurrence with 13 months of follow-up till now, and we are still following-up this patient.

**Lessons:**

MLA is an extremely uncommon malignancy with difficult diagnosis, unclear treatment and poor prognosis. Familiarizing with the clinical features and optimal management of this rare tumor may increase awareness of the disease among clinicians and pathologists, thus avoiding the misdiagnosis and mistreatment.

## 1 Introduction

Mesonephric adenocarcinoma (MNAC) of the female genital tract is an infrequent non–human papilloma virus-related neoplasm, which is considered to originate from the embryonal remnant of mesonephric duct (also known as Wolffian duct).^[1,2]^ MNAC most commonly occur in the lower human female genital tract, particularly in lateral cervix and vagina, while MNAC in the upper female genital tract is very rare, especially in ovary.^[1]^ Mesonephric adenocarcinomas have only rarely been reported to arise in the uterine corpus, but never been reported in the ovary. It was reported that adenocarcinomas of endometrium and ovary shared morphologic, immuonphenotypic, and molecular features with mesonephric adenocarcinoma, but were lack of association with mesonepheric remnants or hyperplasia.^[1,3]^ Hence, mesonephric-like adenocarcinomas (MLAs) are recently suggested to describe these neoplasms that occur in uterine corpus and ovary. Due to the rareness of the disease, there are only six case reports including ten cases of ovarian MLA in the English literature up to now. The optimal treatment of ovarian MLA, especially the effective chemotherapy regimen after surgery remains unclear. The diagnosis is pathologically confused due to its diverse morphological patterns that could be mistaken for benign mesonephric remnants or other malignant neoplasms.^[4]^ Here, we report a very rare case of ovarian MLA, and discuss the clinical characteristics, pathological diagnosis and treatment of this tumor.

## 2 Case report

The patient has provided informed consent for publication of the case.

**Figure 1:**
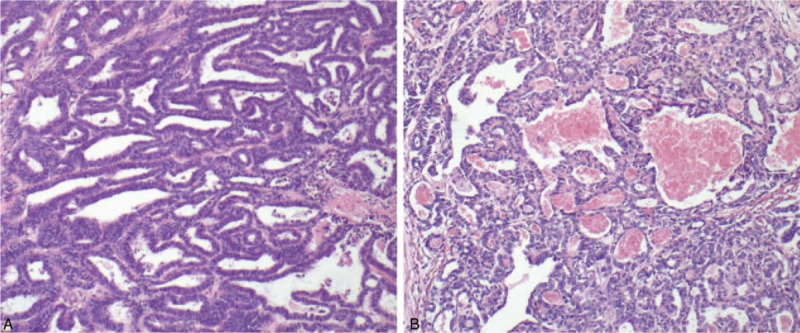
Histologic features of ovarian MLA. Pathological finding revealed the histologic pattern of tumor was glandular, and some of the glands contained luminal eosinophilic material.

**Figure 2:**
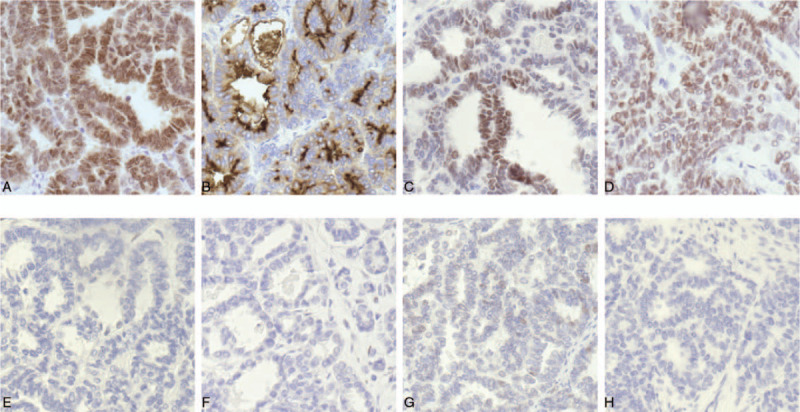
Immunohistochemical staining of ovarian MLA. (A) PAX8 expression in the present case is strong and diffuse, which indicating the tumor arises from the female genital tract. (B) CD10 reveals cytoplasmic and luminal staining. (C) GATA3 positivity is helpful in confirming the diagnosis of mesonephric carcinoma, but the staining may be weak and/or focal. In this case, the staining of GATA3 is a little bit of weak and diffuse. (D) Immunohistochemical staining of TTF-1 is strong and diffuse. Immunohistochemistry showing totally negative staining with ER (E), PR (F), P53 (G), and P16 (H).

A 29-year-old female patient presented to the local hospital with a 20-day history of abdominal discomfort. A transvaginal ultrasound revealed that a solid-cystic mass measuring 10 cm in diameter in the right adnexal area and a cystic mass measuring 5 cm in the left adnexal area. Exploratory laparoscopy was performed, and a 10 cm ovarian solid and cystic tumor with ruptured capsule in the right ovary and a 5 cm ovarian cyst of the left ovary were found during surgery. Then, she underwent laparoscopic right adnexectomy and left ovarian cystectomy. Postoperative pathological results in the local hospital demonstrated the right ovarian tumor was suspected to be a kind of neuroendocrine neoplasm and the left ovarian cyst was benign, hence she was transferred to our institution and pathologic specimens were sent to the pathology department of our hospital. Pathological finding revealed the histologic pattern of tumor was glandular, and some of the glands contained luminal eosinophilic material (Fig. 1), and the immunohistochemical results in our institution showed positive staining for PAX8 (paired box gene 8), CD10, GATA3 (GATA binding protein 3) and TTF-1 (thyroid transcription factor 1) (Fig. 2A–D), while negative staining for ER (estrogen receptor), PR (progesterone receptors), P53, and P16 (Fig. 2E–H). CD10 showed cytoplasmic and luminal staining (Fig. 2B), and TTF-1 positivity was strong and diffuse (Fig. 2D). The final pathologic diagnosis was made to be a primary MLA arising from the right ovary. The patient was a very young woman who had never given birth, and fertility-sparing surgery and comprehensive staging was initially recommended. However, the patient had no desire to give birth and accounted for the rarity and recurrence of the disease, and she finally chose complete staging surgery. A total hysterectomy, left adnexectomy, pelvic plus para-aortic lymphadenectomy and omentectomy were performed for this patient in our hospital, and peritoneal washing was also undertaken during surgery. Intraoperative findings showed no obvious tumor, except adhesion related to previous surgery in the pelvic and abdominal cavity. Pathological examination after surgery revealed the left fallopian tube and ovary were normal grossly and histologically. The cervix and uterine body together measured 9.5 cm in length, and sectioning of the uterus showed a uterine myoma with size of 2cm × 1.5 cm in the posterior wall of the uterus, which was identified to be uterine leiomyoma by histological examination. The cervix was normal histologically and the endometrium was normal with no hyperplasia or malignancy. The pelvic and para-aortic lymph node and omentum were histologically normal with no tumor cell infiltration. The cytology of peritoneal washing was negative.

The patient was discharged 7 days after surgery without any immediate postoperative complications. In accordance with ovarian cancer staging system of the International Federation of Gynecology and Obstetrics (FIGO, 2017), the patient's disease was staged as IC2 (the tumor limited to the right ovary with capsule ruptured before the first surgery). Medical oncology was consulted, and additional chemotherapy was recommended. The patient received adjuvant chemotherapy with four cycles of carboplatin (300 mg/m^2^) and paclitaxel (175 mg/m^2^) 3 weeks after the second surgery. There is no evidence of recurrence with 13 months of follow-up till now. We are still following-up this patient.

## 3 Discussion

Mesonephric adenocarcinoma (MNAC) is a very rare malignant tumor in female genital tract. It is thought to be derived from remnant of the regressed Wolffian duct (also called Mesonephric duct). During embryonic development, the females have two sets of paired primitive reproductive ducts: the paramesonephric (Müllerian) and the mesonephric (Wolffian) ducts. In the embryologic females, Mullerian ducts become female reproductive ducts, while the mesonephric ducts degenerate.^[5]^ However, vestiges of mesonephric ducts may persist along the female genital tract in the form of epithelial inclusions which are called mesonephric remnants.^[4]^ The embryological remnants are found predominantly in the para-ovarian region (epoophoron and paroophoron) and deep in the cervical stroma in the lateral walls. Hence, mesonephric adenocarcinomas in the female genital tract occur most commonly in the cervix and vagina, and less frequently in upper female genital tract.^[6]^

Mesonephric adenocarcinomas have only rarely been reported to arise in the uterine corpus, but never been reported in the ovary. Adenocarcinomas of ovary, which sharing morphologic, immunophenotypic, and molecular features with mesonephric adenocarcinoma, but lacking association with mesonephric remnants or hyperplasia have been reported.^[7]^ MLAs are recently recommended to describe these neoplasms. It was argued whether these cases occurred in the ovaries or uterine body essentially represent mesonephric carcinoma or endometrioid carcinoma that were similar to MNAC, and they suggested these neoplasms could be designated MLAs unless the histogenesis of these tumors is firmly established.^[1,4,7,8]^

Morphologically, both MLA and MNAC could reveal different growth patterns including retiform, papillary, tubular, sex cord, glandular, glomeruloid, spindled, sieve-like, and solid architectures in various combinations, even in the same neoplasms.^[2,7,9]^ MLAs lack mucinous and squamous differentiation and may also show morphology reminiscent of the corded and hyalinized variant of endometrioid carcinoma.^[6,9]^ Immunohistochemically, MLAs frequently exhibit positive staining for calretinin, GATA-3, and CD10 (luminal staining), and almost all of them are positive for TTF-1 and negative for PR/ER. Moreover, the wild-type p53 could also be expressed in MLA.^[2,9]^ Molecularly, MLA, similar to MNAC, is characterized by activating *KRAS* mutations, but lack of *PTEN* mutation/deletion or microsatellite instability and gain of chromosomes 1q. *PIK3CA* mutations, which have never been found in mesonephric adenocarcinoma, were identified in nearly half of MLAs.^[6,10,11]^

There were only six case reports including 10 cases of MLA arising in the ovary in the English literature up to now (Table 1). According to these case reports, MLAs of the ovary mainly occur in middle-aged to older female adults, and the 29-year-old patient in our case was the youngest one in all the reported ovarian MLAs. Among these cases, nearly half of these cases (5 of the 11 cases) were diagnosed at a late stage (stage III–IV), but fortunately 4 of the 11 cases were stage I. There was only one patient with bilateral ovarian MLA and the other patients were diagnosed with MLA occurred in one ovary. Blood tumor markers such as CA125 were not reported in most of the case reports. Limited data from the table showed elevation of serum CA125 could be detected in a late stage of MLA (stage IV), but it may be in normal range in an early stage (stage I in the present case).

**Table 1:** Clinicopathologic features of mesonephric tumors in the ovary.

Cases reported	study	Age	Site	CA125^∗^ (U/mL)	FIGO stage	Surgery	Chemotherapy	Duration of follow-up	Alive/expired
5	Marie McFarland et al ^[7,10]^^†^	54–72	One ovary (two cases), both ovaries (two cases), unknown (one case)	NA	IA (three cases) IB (one case) IIIC (one case)	HYS+BSO (three cases), BSO (one case), Unknown (one case)	NA	1–18 months	Alive
1	David B. Chapel et al ^[12]^	80	One Ovary	1077	IV	HYS+BSO	PC	3 months	Alive
1	Jennifer Pors et al^[2]^	67	Ovary (unknown)	NA	IC	NA	NA	NA	NA
1	W. Glenn McCluggage et al^[3]^	61	One ovary	NA	IIIA	HYS+BSO	PC (6 cycles)	NA	NA
2	Brie Kezlarian et al^[1]^	3645	One ovary(Both in one ovary)	NA	IIIIIIA	NA	NA	NA	NA
1	Present case	29	One ovary	13 (In normal range)	IC	HYS+BSO	PC (4 cycles)	13 months	Alive

Ovarian MLA has no specific clinical manifestation, and there might be some symptoms of abdominal distension or pain when tumor in the ovary is large enough to cause abdominal discomfort. Hence, it is very difficult to detect early stage ovarian MLA just like any other ovarian tumors. The only way to detect the disease at an early stage is through routine physical examination like the patient in this case. Further examinations, such as serum tumor markers, CT (Computerized Tomography) or/and MRI (Magnetic Resonance Imaging) and PET/CT (Positron Emission Tomography-Computed Tomography), are required when a routine abdominal ultrasound examination reveals a mass in the adnexal region. Blood tumor marker test may detect an elevation of CA125 in patient with ovarian MLA. Therefore, preoperative diagnosis of ovarian MLA is extremely difficult and definite diagnosis mostly depends on postoperative pathology and immunohistochemistry. The differential diagnosis of MLA should include endometrioid adenocarcinoma, and serous adenocarcinoma. Endometrioid adenocarcinoma is characterized by squamous, mucinous, or ciliated differentiation and presence of precursor lesion (endometrial intraepithelial neoplasia/atypical hyperplasia), and expression of ER and PR but it is negative for GATA-3 immunohistochemically. Serous carcinomas have high-grade nuclear atypia, pronounced pleomorphism, and are associated with abnormal p53 staining and typically strong and diffuse p16 staining. Serous neoplasms are immunoreactive for WT1 (Wilms’tumour 1) and negative for GATA-3, whereas MLA will exhibit the opposite staining pattern.^[1,6]^

The pathogenesis of these tumors, which have been termed as “mesonephric-like adenocarcinomas” is unknown. Hence, for pathologists it has been debated whether these represent endometrioid adenocarcinomas that closely mimic mesonephric adenocarcinoma or mesonephric adenocarcinomas that occur in the ovary/endometrium. However, it is not so important whether the rare disease should be diagnosed as ovarian MNAC or MLA for the gynecologic clinicians, because the treatment for these neoplasms, which the gynecologic oncologists are more concerned about, may be the same. Due to the paucity of available data related to treatment of the rare disease, there is no established guideline about standard therapy of ovarian MLA. Although there is no guideline for the rare malignancy disease, comprehensive staging surgery may be the initial choice for localized disease. A hysterectomy, bilateralsalpingo-oophorectomy, pelvic plus para-aortic lymphadenectomy and omentectomy were performed in most of the previous cases (Table 1). In our case, the patient was a very young woman who had never given birth and the FIGO stage is IC2, and fertility-sparing surgery and comprehensive staging were initially recommended. However, the patient had no desire to give birth and accounted for the rarity and recurrence of the disease, and she finally chose complete staging surgery. Chemotherapy is also very important and necessary treatment method for ovarian MLA like any other ovarian cancer. Up to now, there is also no reported research about which chemotherapy regimen is most effective or sensitive to MLA. There were only two case reports mentioning chemotherapy regimen after surgery, and chemotherapy with carboplatin plus paclitaxel were adopted in both of the two cases (Table 1). Given this, we use conventional chemotherapy drugs, carboplatin plus paclitaxel, to treat this patient. The patient remains with no evidence of disease recurrence with 13 months of follow-up, indicating that complete staging surgery followed by combination chemotherapy with carboplatin plus paclitaxel are very effective in the treatment of ovarian MLA. A recent study showed a recurrent MNAC case with good response to combination chemotherapy with carboplatin plus paclitaxel.^[13]^ These evidences suggest that combination chemotherapy with carboplatin plus paclitaxel may be the effective chemotherapy regimen to treat MLA like other ovarian cancer, and could be recommended as a first-line chemotherapy regimen.

We presented a case of ovarian MLA without sign of relapse or metastasis so far, which revealed a good response to combination chemotherapy with carboplatin plus paclitaxel. Due to the chemosensitivity, we think it is plausible that chemotherapy with carboplatin plus paclitaxel plays a major part in MLA. In addition, a recent study has shown the vast majority of MNAC/MLA harbor *KRAS/NRAS* mutations, inhibiting the RAS/MAPK pathway could potentially be a useful method in the treatment of ovarian MLA.^[14]^ However, further studies will need to be conducted to better understand the clinical characteristics, optimal treatment and prognosis of MLA.

## 4 Conclusion

Mesonephric-like adenocarcinoma is an extremely rare malignant tumor in the female reproductive system, which is believed to arise from the embryonal remnants of mesonephric ducts. Preoperative diagnosis of MLA is exceeding difficult, and postoperative pathological diagnosis is challenging since MLA typically shares a mixture of morphologic patterns. Comprehensive staging surgery followed by systemic chemotherapy may be the initial choice for localized disease. Patients with ovarian MLA may be sensitive to chemotherapy drugs with carboplatin plus paclitaxel, which could be recommended as a first-line chemotherapy regimen. Further studies will need to determine the exact role of chemotherapy and select better therapeutic schedule.

## Author contributions

**Conceptualization:** Qiuhe Chen, Chuan Xie.

**Data curation:** Qiuhe Chen, Yangmei Shen, Chuan Xie.

**Formal analysis:** Qiuhe Chen.

**Investigation:** Qiuhe Chen, Chuan Xie.

**Methodology:** Yangmei Shen, Chuan Xie.

**Software:** Qiuhe Chen, Chuan Xie.

**Supervision:** Qiuhe Chen, Chuan Xie.

**Writing – original draft:** Qiuhe Chen, Chuan Xie.

**Writing – review & editing:** Qiuhe Chen, Chuan Xie.
